# Research Translation and Emerging Health Technologies: Synthetic Biology and Beyond

**DOI:** 10.1007/s10728-016-0334-2

**Published:** 2016-12-09

**Authors:** Sarah Chan

**Affiliations:** 1 Usher Institute for Population Health Sciences and Informatics, University of Edinburgh, Nine Bioquarter, 9 Little France Drive, Edinburgh, EH16 4UX UK; 2 Manchester Synthetic Biology Research Centre, University of Manchester, Manchester, M1 7DN UK

**Keywords:** Synthetic biology, Research translation, Research ethics, Health technology, Experimental therapy, Medical innovation

## Abstract

New health technologies are rapidly emerging from various areas of bioscience research, such as gene editing, regenerative medicine and synthetic biology. These technologies raise promising medical possibilities but also a range of ethical considerations. Apart from the issues involved in considering whether novel health technologies can or should become part of mainstream medical treatment once established, the process of research translation to develop such therapies itself entails particular ethical concerns. In this paper I use synthetic biology as an example of a new and largely unexplored area of health technology to consider the ways in which novel health technologies are likely to emerge and the ethical challenges these will present. I argue that such developments require us to rethink conventional attitudes towards clinical research, the roles of doctors/researchers and patients/participants with respect to research, and the relationship between science and society; and that a broader framework is required to address the plurality of stakeholder roles and interests involved in the development of treatments based on novel technologies.

## Introduction

For almost all areas of bioscience, one of the spheres of application that holds most promise and interest is the potential to develop new therapies to improve human health. The current biotechnological landscape features a proliferation of possibilities for new health technologies from areas such as genomic and personalised medicine, gene editing, reproductive technologies, stem cells and regenerative medicine.

Ethical concerns associated with these technologies often attach to the social consequences of their eventual widespread use: for human welfare and global health equity; for our definitions of health, wellbeing and disease; even for our definition of “being human”. These issues, however, are not the beginning of the story: important questions arise even before these technologies reach the point of clinical availability and wider health impact, in terms of what is required to develop treatments to that point, and the ethical issues associated with the use of experimental therapies and participation in clinical research.

Synthetic biology offers a particularly interesting example in this respect because of its fluidity and ‘multipleness’ as a concept [10]: it encompasses a diverse range of possible technologies while simultaneously functioning as an object of expectation, “the new technoscience”, around which “socio-technical imaginaries” and promises of future benefit, including better health, are built [1]. Given the extent to which the field has been constituted around these possibilities, thinking about synthetic biology as the focus of promissory narratives rather than as a single field of research allows us to shift our attention from technology-specific concerns and towards factors relevant to the social shaping of health innovation in general.

My aim in this paper is therefore to examine issues related to the development of novel health technologies as experimental treatments, using the idea of synthetic biology health technologies to illustrate features of the health innovation landscape that require ethical attention. I start by identifying a number of factors to be taken into consideration when thinking about the development of experimental treatments and new therapies, focusing on ethical issues surrounding participation in clinical research, and to the clinical use of experimental treatments (which may or may not be the same thing, a point which I will return to discuss later). Considering the likely pathways by which synthetic biology and other novel health technologies may develop to the point of clinical application, and the problems that arise from this, I argue that existing ethical approaches to clinical research participation may no longer be appropriate to describe the relationships and interests of stakeholders in this process: clinical translation in the contemporary setting poses concerns that require us to rethink our ethical approach. I outline an alternative approach based on conceptualising science as a social institution of public benefit, with the aim of shifting ethical thinking and practice at the research translation interface.

Finally, on the basis of this, I will draw some tentative conclusions about the conditions by which research into emerging technologies for human health and the development of novel therapies should proceed and how it should be regulated. I suggest that greater openness in relation to research, global cooperation with respect to research and to health care, and the mechanisms for accessing therapies are three important facets that should be addressed.

## Synthetic Biology and Health Technologies

Synthetic biology has been broadly described as “a field which aims to construct living systems de novo” [10]. One manifestation of synthetic biology that has received considerable public attention is the work carried out by scientists at the J Craig Venter Institute, whose creation of a bacterium with a synthetic genome [25] was widely reported as “the first artificial life form”. Although this did not really constitute ‘creating life from scratch’ as such [32, 50], the event served to bring synthetic biology into the spotlight, including prompting an investigation and report by the US Presidential Commission for the Study of Bioethical Issues [26, 47].

While this minimal genome synthesis research is a high-profile aspect of synthetic biology, the field is broader than this. Its activities have been categorised into three general approaches: engineering of modular biological parts that can be combined into cellular systems for various purposes; cell engineering via genome synthesis (including the Venter Institute work); and protocell creation [45]. These diverse and varied endeavours are united by the aim of ‘engineering life’ or, as scientists engaged in the field have described it, “the design and construction of new biological parts, devices, and systems and the re-design of existing, natural biological systems for useful purposes” [10]. Nevertheless, scholarship in science and technology studies has highlighted the ways in which the ‘field’ of synthetic biology resists clear definition, even as it emerges from a combination of certain sets of practices, shared goals and epistemologies, networks of people and institutions, and socio-political positioning [1].

Considerations of the ethical challenges posed by the potential application of synthetic biology for human health therefore attach to the idea, more than the everyday reality, of what synthetic biology ‘is’. While the possibilities for health technologies from synthetic biology are myriad—for example new systems for drug development and production; engineered biological circuits; biosensors; cell-based therapies; microbiome engineering—it is the idea of synthetic biology as the focus for socio-technical imaginaries that attracts concerns. In this regard, the concept of “synthetic” in the sense of ‘artificial’, in opposition to ‘natural’, has emerged strongly as a feature of synthetic biology. Other key characteristics of synthetic biology are the importance of design and engineering principles, whether it be in re-designing existing systems and organisms or designing new ones ‘from scratch’; and the idea of improving on nature through this process.

Each of these conceptual features gives rise to potential ethical issues: Incorporating synthetic biology components into medical treatment could result in hybrid humans whose bodies are a combination of ‘natural’ and ‘artificial’ biological components, which in turn might have implications for our understanding of embodiment and its relationship to the ‘natural’ or ‘biological’. The reductionist approach to biological systems might also prompt us to ask whether ‘humanity’ is something that can or should be designed or engineered, or whether something is lost in the apparent reduction of life to machine [17, 19, 33]. Another possible consequence of synthetic biology therapies is that, as for other forms of regenerative medicine, they might have the potential to cure or repair our bodies to the point of being ‘better than well’ [22]. This of course invokes questions related to the ethics of human enhancement, including the expanding boundaries of what is considered to be disease, what is in consequence viewed as therapy, and hence the ‘moving goalposts’ of medicine.

Analysis of these complex issues will need to take into account the construction of moral concepts in relation to biology, for example how concepts such as ‘synthetic’ and ‘natural’, rather than being immutable categories, are actively constituted, negotiated and re-valued through scientific practices and in relation to scientific ideals [10].

My concern in this paper, however, is not with these further-off questions about the eventual use of synthetic biology health technologies once developed, but in using the ‘imaginaries’ of synthetic biology to scrutinise more closely the process of research translation, from the promises generated by bioscience innovation, to the testing of potential new therapies, and how and to whom these emerging treatments are made available.

## Developing Synthetic Biology Therapies: Some Possibilities

The ways in which new health technologies are currently emerging reveal social trends and attitudes to novel treatments that allow us to speculate about how synthetic biology therapies might developed. By this I mean not speculation regarding the technological or scientific possibilities, but an exploration of the factors that are likely to drive the process of development and influence the reception of these therapies. Health technologies such as stem cell treatments and genomic testing that are presently in the transition stage between novel experimental therapy and standard medical treatment provide us with models for how new fields of research can translate from the laboratory to the clinic and highlight potential problems, while established models such as the pharmaceutical industry also highlight relevant factors that may influence future health innovation.

Thinking about likely scenarios in relation to the evolution of synthetic biology treatments will help to identify factors that characterise the landscape of health innovation, before proceeding in Part 4 to explore the implications and issues raised. Addressing these issues will uncover underlying ethical tensions that not only may arise with respect to the future development of synthetic biology therapies, but are also relevant to the health technologies of today.

Synthetic biology health technologies, then, are likely to:

- *… be sought*-*after by patients* Synthetic biology therapies may offer the possibility of new and more effective treatments for disease, or even therapies for previously untreatable conditions (not to mention their potential for enhancement uses). As such, they will be desirable to some even at the experimental stages. Patients suffering from the burden of chronic disease that can be only incompletely relieved with current treatments, or those for whom no other treatment option is available, or those for whom experimental treatment may be a last-ditch attempt: all of these are situations in which people may wish to avail themselves of emerging treatments even if not yet fully established. The rapid growth of the “stem cell therapy” industry [36, 38, 49] whereby numerous clinics around the world are “exploiting patients’ hopes by purporting to offer effective stem cell therapies for seriously ill patients, typically for large sums of money, but without credible scientific rationale” [30], is evidence of a large unmet demand for new therapies.
- Whether novel treatments either will or should be made available in such circumstances of demand, and under what conditions, is a matter that requires careful consideration. The role of marketing in promoting new health technologies and making them attractive to the public is another element that cannot be ignored and that comes into play particularly when commercial interests are involved. Both of these issues will be discussed further below.

- *… involve different types of risk* The clinical research needed to develop synthetic biology health technologies may require a different approach to assessing and managing risks. Because of the range of technologies encompassed by synthetic biology, the possible risks are diverse in nature, as well as level and scope. For example, treatments that make use of engineered micro-organisms may have effects not just for individual patients, but at environmental or population level. Additionally, due to the relative novelty of synthetic biology as a field of science as well as its application to health care, the risks involved represent something of a qualitative as well as quantitative unknown: there will be uncertainty over not only the likelihood but the nature of potential adverse effects. This may have consequences for how we should approach such technologies at the clinical research stage and in the long-term.

- *… develop at different rates across countries* The pace at which synthetic biology therapies are developed, tested and become available is unlikely to be a uniform one, internationally (and even nationally) speaking. Transnational differences in research capacity and in particular the regulation of research and health care will most probably lead to differential rates of progress with respect to the development of therapies, and to discrepancies in the local availability of treatments once established. That this is already the case with respect to virtually every other health technology is evident merely by reference to differing standards of global health care, as well as with respect to more specialised technologies and the phenomenon of medical tourism produced by discrepancies in treatment availability between regions [18].
- These differences will mean that both at the experimental stage and later, access to synthetic biology therapies will likely vary amongst countries, with treatments being available in one country but not another. Research would also be unevenly distributed; however, increased local research contribution would not necessarily correlate with increased local availability of treatments. This raises a number of important ethical problems in terms of global justice and transnational regulation of health technologies, as will be discussed below.

- *… be driven partly by commercial concerns* Commercialisation has become an integral part of the biotechnology innovation pathway. While there may still be particular instances of health technologies that are developed primarily through public research, very few if any fields of biomedical science are completely devoid of commercial interests. Synthetic biology is no exception: already the push to secure patent rights over various aspects of the technology has begun, sparking debate as to what the effects of intensive patenting in this area are likely to be, and whether the current patent system is in fact an appropriate or optimal mode of managing intellectual property in this area of research [9, 13, 52].
- As synthetic biology research progresses towards clinical application, we can expect to see further debates over intellectual property rights, similar to those currently occurring in the areas of stem cell research [42, 49], genetic testing [16, 59] and gene editing [58]. There are likely also to be consequences for how these technologies are made available to the public, in terms of access and cost; and how they are made *desirable* to the public, particularly through marketing, both direct and via the health care system.

## Issues of Concern

The features of the probable developmental pathway for synthetic biology health technologies as outlined above give rise to a number of particular concerns. While none of these are unique to synthetic biology, together they highlight what I would argue is an overall need to reconsider the ethical paradigms that apply to clinical research and experimental treatment, and to science and medicine in general, in prospect of the emergence of new health technologies including synthetic biology treatments.

### Transnational Challenges and Synthetic Biology Tourism

Transnational discrepancies in the regulation of research and health care and hence the availability of treatments at the early stages of development are likely to result in both health and research tourism with respect to synthetic biology technologies.

A textbook case for this in recent times is embryonic stem cell research, the moral controversy over which has produced an international patchwork of regulation ranging from the highly restrictive to the permissive, and in some cases still no regulation at all [31]. In the case of stem cell science, transnational discrepancies in policy have led to research tourism, regulatory uncertainty and problems for international scientific cooperation [31, 41, 43]. While the ethical issues and consequent regulatory concerns associated with synthetic biology have not (so far) polarised public opinion to quite such an extent, it is likely to be differences in the regulation of health care research and clinical trials, rather than basic research, that come to bear in this case.

Health care, provision of medical treatment and experimental therapies, and clinical research are much more tightly regulated in some countries than others, and this will open up disparities as synthetic biology therapies begin to emerge. If regulations are too restrictive (or perceived as such) in one jurisdiction, scientists and biotech companies may simply relocate their research activities to another, more permissive jurisdiction. This will have economic and political implications: concerns over falling behind on the scientific front and the loss of economic value to local biotech industry will be juxtaposed against potential ethical objections and concern for proper regulation. Issues may also arise with respect to how the benefits of research are or should be made available: missing out on the research may mean reduced access to its fruits, but on the other hand, to reap the benefits while avoiding the ethical and resource burdens of carrying out the research creates problems of justice and even complicity.

Further complexities emerge when clinical research is carried out across national boundaries, a situation that is facilitated and encouraged by transnational regulatory differences—when, let us say, the knowledge base, research capacity and economic investment belong to one country, but the clinical trials are conducted in another. This problem has received most attention in the context of cross-border research in the developing world (see for example [2, 20, 23]), but the issues it creates do not fall out solely along the so-called North–South axis.

Health tourism is another, though not unrelated, phenomenon that can create regulatory and ethical dilemmas [18]: if treatments are not available in one place, whether because of resource limitations or regulatory obstacles, people will seek to travel elsewhere to obtain them. This may have ramifications for local health care systems if unexpected side-effects or adverse consequences of ‘rogue’ treatments subsequently require medical attention. On the other hand, if a treatment is showing signs of success but has not yet been approved in a given country, or is unavailable for other reasons, then to allow access only to those who can afford to travel elsewhere to receive treatment creates potential problems of justice. This type of situation is already emerging with respect to stem cell therapies, where medical tourism has been identified as a phenomenon of concern [21, 40, 53], as well other health technologies including reproductive medicine [5, 46] and assisted dying [44] (if the latter can be classed as a ‘health technology’, that is).

To address the issues of both research and health tourism in relation to new health technologies, a concerted effort towards global cooperation will be required from policy-makers and from the scientific and medical community. This need not mean complete regulatory harmonisation—indeed, complete harmonisation may not be the best way to serve diverse global populations with differing health needs—but at the very least, an awareness of the international context and the effects of local regulatory and technological developments is necessary.

### Commercialisation and Consumerism

The almost inevitable involvement of commercial interests in the development of synthetic biology therapies will have far-reaching consequences. One, highly likely, is that in order to protect commercial interests, some may seek to assert intellectual property rights over various aspects of synthetic biology research.

The effects of IP, particularly the patent system, on scientific progress and access to innovation have been much-discussed [35, 59], and these issues are likely also to apply in the case of synthetic biology. Without recapitulating these debates, however, there is another aspect of commercialisation which merits discussion: the expectation of profit as an incentive for research into synthetic biology therapies creates pressure to ensure a receptive market for these therapies. Such pressure will encourage the active marketing of synthetic biology health technologies, which will in turn influence public expectations, understanding and attitudes towards these technologies and synthetic biology in general. This will have a profound effect on how research is driven and how participant interactions with technology are mediated—for example, through the health care system, by biotech companies or by patient lobby groups.

One scenario that might conceivably occur is the direct-to-consumer (DTC) marketing of new synthetic biology therapies. In the context of genetic testing, DTC provision has been a source of ongoing ethical consideration [28, 29]; DTC marketing of stem cell therapies has likewise provoked recent concerns [36]. Similar issues would arise if DTC synthetic biology therapies became a reality. Some of the fears about DTC health technologies relate to availability without oversight: unregulated provision means that harms may be more likely to result and risk cannot be managed effectively. For synthetic biology therapies, this would echo concerns expressed about synthetic biology research and ‘backyard bioengineering’ or ‘DIY science’ [47]: a coordinated approach is necessary, to health applications as well as research.

Another risk is that public expectation, assisted by profit-driven marketing, may outpace the reality and the actual therapeutic capacity of the technology. Again, the development of stem cell therapies aptly illustrates this possibility [36, 49]. This may lead to nothing worse than disappointment and public disillusionment with synthetic biology if promised outcomes fail to manifest—indirectly harmful nonetheless, as the erosion of public trust in science will have long-term adverse consequences for science and society alike. Alternatively, however, it may result in direct harms ranging from mild to serious, if hopeful patients are led to squander money on ineffective treatments, or if inadequately-tested treatments pose a danger to health and life.

In either case, allowing profit to be a major driver of synthetic biology health research will almost certainly have knock-on effects on users’ understanding of the underlying science, the risks involved, the implications and their expectations of successful therapy. Commercial pressures on technology will thus change the relationships between developers, providers and users of synthetic biology therapy, as well as the terms under which people may choose to take up these treatments. While this is not to say that commercialisation ought to be avoided altogether, an awareness of these factors is crucial and their effects must be carefully scrutinised.

### Risk and Access to Experimental Therapies

We noted previously that synthetic biology therapies may involve a different kind of risk to that usually encountered in clinical research, due to the diversity and novelty of the technologies involved, but that they will also be desirable to some despite this. As synthetic biology therapies enter the testing phase, patients may wish to enter trials in order to have the chance of receiving potentially beneficial new treatment. This raises a broad problem of clinical research and medical ethics: under what conditions should patients be given treatments that are still at the experimental stage, even at their express request? Or, to cast it in another light, under what conditions should willing volunteers be permitted to take part in clinical research? The range of circumstances under which patients might wish to take part in trials of synthetic biology therapy, or to receive experimental treatments, might be broader than the range of circumstances under which medical practitioners are able to administer treatment, or researchers able to admit them to trials.

Clearly it would be nonsensical to say that patients should be entitled to receive whatever treatment they demand, or any volunteer, no matter how unsuitable, should be included in a clinical trial. It is also the case, however, that not all of the limitations on the availability of experimental treatment and the exclusion criteria for clinical trials necessarily serve the interests of doctors or patients, researchers or participants, medicine, science or the public: some may instead be directed at limiting liability, avoiding negative press or expediting the progress of a therapy to market [15, 60]. In a situation where a patient/participant is willing to take part in research, a clinician judges that it might be in their medical interests to undergo the procedure or receive the treatment, and scientifically useful information may be gained, what reasons are there to prevent this taking place? How much risk, and what uncertainties with respect to risk, should be tolerated, and who should decide this? When taken in combination with the possibility of not only health and research tourism but research *participation* tourism, the issue of access to experimental therapies and participation in clinical research becomes more pressing.

The mitigation and management of risk becomes an important factor in this regard, and one that invokes societal-level considerations of regulation and mechanisms of ethical research governance. It is not only individual patients/participants and clinicians/researchers who are involved in decisions over experimental treatment, but policy-makers who set guidelines as to what sorts of research is permitted and ethics committees who interpret these guidelines. Addressing this issue thus requires us to consider, in addition to the roles and relationships of individual actors in specific cases or circumstances, the relationship between science and society and how this is mediated through the regulation and governance of research.

I and others have argued elsewhere, taking the example of cancer drug trials [15, 60], that ethical attitudes towards participation in ‘risky’ research may be overly paternalistic and skewed in favour of ‘protecting’ potential participants. Although great emphasis in research ethics is placed on informed consent, autonomy and voluntary choice, there are many potential trials in which patients are never given the chance to consent and cannot exercise any autonomy to make a choice about taking part, because the research is not permitted or would not receive ethics approval. Thus participants’ autonomy is respected as far as saying ‘no’, but not as far as saying ‘yes’—an inherent asymmetry in the paradigm of research ethics.

If we are going to uphold, on the basis of respect for autonomy, the right of individuals to refuse to take part in research for whatever reason (no matter how irrational or against their medical interests) then we ought also to consider whether respect for autonomy might also support some sort of right to be included. At the least, respecting autonomy in relation to research should prompt us to consider a role for potential participants to have some input into the research process and into decisions over what sorts of research should go ahead—to be part-owners and active stakeholders in research. We must also ask what levels of knowledge and engagement are necessary for participants to be able to exercise genuine autonomy as active co-producers of research, rather than passive subjects; and in turn what will be required to achieve this.

## Discussion

The issues identified above are not discrete but are interlinked aspects of a changing, sometimes ill-charted technosocial landscape. Together, they suggest that a broader, more joined-up approach to ethics across health care, clinical research, biomedical science and biotechnology is required to deal with the health technologies that are emerging at the intersection of these areas, including synthetic biology. Before discussing how this might be achieved, I wish to address one further issue that also highlights the need for a new approach.

### Clinical Research or Experimental Treatment?

“I continue to be astounded by how little the medical profession knows about my condition. I guess that’s why they call it the practice of medicine” [24], at 105.

Thus far, in discussing the transition phase of synthetic biology therapies from laboratory to clinic, I have considered clinical research and experimental treatment together, and although distinguishing the two by the use of different terms, I have not examined whether there may be material differences in the ethical implications of each. I noted earlier, however, that this was an issue remaining to be resolved: are experimental medical procedures and clinical research the same thing or not? The problems of ethics and regulation encountered in this ‘grey area’ might also be tractable to a different way of thinking about biomedical research and health care, as aspects of the same broader enterprise.

Research and treatment are often regarded as different kinds of activities, subject to different ethical norms [37]. In general, innovative treatment is characterised as health care and the (medical) best interests of the patient are the dominant consideration, whereas the focus in clinical research is much more on informed consent as the keystone of ethical practice—sometimes to the exclusion of other considerations such as harm [55]. But why should this be so? What is it about the research context that warrants this shift in ethical approach and underlying assumptions? When does a novel therapy cross the line between research and treatment—science and medicine—and where is that line to be drawn?

There is an argument for considering medical practice and medical science as aspects of the same endeavour [34]. Conceptually, the difficulty of separating one activity from the other perhaps indicates that they are not so different, while logically, there is little to indicate why they should be. Even areas of established medical practice can have an investigative aspect or lead to new scientific insights: recent moves, for example, towards making patient health information available for research purposes illustrate that data gathered in the context of medical practice can be of immense value for medical science. And of course one of the ‘side effects’ of taking part in research trialling new therapeutic agents is that the treatment might actually work! That is to say, participation in research can be of direct therapeutic benefit to the participant. Certainly this is not always going to be the case, and caution is needed to manage possible misconceptions on the part of would-be patients. The possibility of medical benefit from research participation is, however, if not a secondary aim then at least not an unforeseen consequence of clinical research.

This argument gains further purchase when we consider the factors outlined above. Consider the person attending a health care clinic who hears about a new form of therapy privately available from a biotech company, decides to pay for the treatment that is then administered by her doctor, but allows health monitoring and data collection for research purposes. Is she a patient, a research participant, a consumer or all three?

The problem of distinguishing research from medical treatment illustrates that end-users have multiple roles with respect to technology: patient, participant, consumer. In some ways this is not a new thing—but the current manner in which new health technologies (including synthetic biology) are emerging intensifies the ethical tensions produced by this plurality of roles, and the multiplicity of stakeholder interests. The blurring of distinctions between research and treatment and the increasing integration of these activities within the health system requires new modes of ethical and regulatory thinking [34].

With respect to research participation, for example, we should allow that many would-be participants will be motivated by benefit as well as beneficence. To take adequate account of this, we need a conceptual framework for the ethical governance of research that acknowledges and supports participants’ interests not only in being protected from possible harms caused by the use of treatments at the experimental stage, but in receiving the benefits that such treatments might also produce. At the same time this framework must recognise the value of the information gathered in the course of administering such treatments at all stages of development: not just at the clinical trials phase but through what might usually be deemed ‘experimental treatment’ and even beyond, as such therapies enter the realm of standard medical practice. It should therefore recognise and support the public interest, and the interests of scientists and future patients, in making such information available for research use.

### A Conceptual Approach to Science Ethics and Research Translation

I have argued that to meet the challenges posed by the emergence of synthetic biology health technologies there is a need for a paradigm of science ethics that can support new ethical understandings of clinical research, the practice of medicine, the roles of researchers/clinicians and participants/patients and the relationship between science and society, as they are likely to evolve with respect to emerging health technologies. While to describe the elements of a possible framework in detail is beyond the scope of this paper, I outline here an approach that may provide a foundation for further development.

Central to this broad approach to science ethics is an understanding of science as a social institution that is of public benefit. In characterising science as a public good and an institution that is constituted and supported by society, I hope to suggest a moral perspective on science that can incorporate research ethics, medical ethics and bioethics alike and allow consideration of both individual and societal concerns. A few of the implications of this approach would be as follows:

If science is a public institution, scientists should perhaps be regarded as public officers who have broad social responsibilities with respect to their field of endeavour. Failures of social responsibility should be viewed in the same light as breaches of scientific integrity, both being derelictions of a scientist’s public duty. Giving scientists the responsibility and the opportunity to be stewards of their own specialised knowledge for the public’s benefit may be an effective way of addressing some of the problems associated with regulation of science and biotechnology: for example, scientists are among the best-placed to promote global cooperation and transnational harmonisation of health technologies at the experimental stage.

Lest it seem that this interpretation of the nature of science places too heavy a burden of responsibility on scientists, the public also have corresponding duties with respect to the social institution of science. It has been argued elsewhere [11, 27, 51] that research participation, and supporting science through participation, may be a moral obligation. Others have contested this, particularly with respect to what constitutes a moral obligation or duty [6–8, 56], but the thrust of the original arguments is that, even if it falls short of an obligation, participating in research is something that is ‘good to do’. This makes sense if we consider science as a whole, not just individual research projects, to be a public good and a valuable social institution [12, 14]. While any sort of compulsion, even a weak moral one, to take part in research seems antithetical to current principles of research ethics, the notion may be more amenable if we understand it as a more democratic duty to participation in research broadly construed: not just to be an experimental test subject but to support, contribute to and have a say about research. Fulfilling this obligation would also entail a duty to be informed and to engage actively with science.

Might the same understanding of science as social institution and research as a partly democratic social process in addition support a right (also broadly construed) to participate in research? In the context of experimental treatments, the recent turn towards the language of rights and rights-based claims as attempts to gain access to treatment may be considered ethically problematic: for example, recognising and granting an unfettered ‘right-to-try’ in relation to experimental treatments might have rather undesirable and unethical effects on patient and public health, and on health technology innovation.

Rather than focusing solely on access to the end-products of science at the later stages of clinical innovation, however, we should take a wider view of what constitutes research for the purposes of understanding a right to participation. Once again, stem cell science provides an illustration: in the turbulent environment of market-based supply of innovative therapies and health consumer demand, scientists warn that giving into what they see as unreasonable and ill-founded demands by patients will lead to exploitation and harm [3, 4, 39]; patients, meanwhile, have developed a deep mistrust of the dominant institutions of science and mechanisms for therapeutic innovation, from which they feel excluded [54]. Is it perhaps a lack of opportunities for democratic participation and engagement with both basic and clinical research, upstream as well as downstream, and with the governance of science and innovation, that has led to this impasse?

A right to participate in research broadly conceived, therefore, would not imply a right to receive whatever experimental treatment one desired or to compel scientists to conduct whatever research we might want to take part in, but rather a right to take a more active role in research than the current, very limited, right only to refuse what is offered us. We might, indeed, view both the right and the duty to participate in research as an aspect of scientific citizenship, and in exercising that right and fulfilling our civic duty, we would become active participants rather than passive subjects.

Lastly, the public interest in science and the rights and duties that flow from this rely on the assumption that research and the fruits of research will be used for public benefit. As we know, however, this is not always the case, particularly when it comes to private profit-driven science. The privatisation of science should therefore be looked upon with the same scepticism as the privatisation of other services seen as essential to a functioning society. Properly managed, public/private partnership in science can promote efficiency to mutual benefit, but we must be vigilant of allowing private interests to subsume those of the public.

## Conclusions: Science Ethics and Research Translation

In this paper I have identified some ethical issues that may arise in the course of development of synthetic biology therapies as an example of novel health technologies, and argued that a new, broader framework for science ethics may be necessary to deal with the challenges that synthetic biology therapies and other emerging health technologies will present. I began by speculating about the possible future evolution of synthetic biology therapies; I will conclude by briefly stating some thoughts to bear in mind as we attempt to guide this evolution. Based on the challenges identified and the account of science outlined above, the following are some of the areas that require most pressing attention:

To overcome potential problems of health and research tourism that may result from transnational regulatory and research differences, global cooperation will be required. An important factor in achieving this will be the power of scientists and the international scientific community to set internal standards of conduct and to instigate their own procedures to maintain these, and they have a responsibility to society to do so.

Access to synthetic biology therapies at stages from early experimental to established treatments will remain a possible source of contention. The interplay of commercial, individual, public and scientific interests here creates complex ethical tensions that will need to be resolved in order to shape synthetic biology research and innovation in the way that most serves the public interest. In particular, the potential effects of commercialisation to limit access and to sway societal attitudes to synthetic biology therapies, neither in ways necessarily conducive to public benefit, must be carefully assessed, and mechanisms to ensure appropriate access (and discourage inappropriate access) to technology emplaced.

Finally, in relation to all aspects discussed: global cooperation, access to the benefits of technology, and the factors that influence public opinion and understanding of synthetic biology, strategies for promoting greater engagement and openness with respect to research will need to be developed. Knowledge, as well as application, is a product of science and therefore should be made available and used for public benefit; and in order to make informed decisions about health technology and to engage adequately as scientific citizens, members of the public must have access to the information needed to develop an understanding of the science. Transnational sharing of knowledge capital will also help to promote global scientific cooperation and achieve greater parity in synthetic biology research. The principle of openness and the concept of scientific knowledge as a public good, to be shared for public benefit, should guide our ongoing considerations in this regard, both with respect to synthetic biology therapies and basic research.
